# Go&Grow: A Social Exergame to Promote Well-Being Among Dementia Caregivers

**DOI:** 10.1093/geroni/igaa057.1840

**Published:** 2020-12-16

**Authors:** Xin Yao Lin, Herman Saksono, Elizabeth Stowell, Margie Lachman, Carmen Castaneda-Sceppa, Andrea Parker

**Affiliations:** 1 Brandeis University, Waltham, Massachusetts, United States; 2 Northeastern University, Boston, Massachusetts, United States; 3 Georgia Institute of Technology, Atlanta, Georgia, United States

## Abstract

Dementia caregivers are at risk for poor health outcomes due to high stress and little time for health-promoting behaviors. The current study examined whether Go&Grow, an exergame (digital game in which play involved real-world physical activity), could increase caregivers’ physical activity and social contact and reduce their stress. Go&Grow allows participants to grow flowers virtually as they increase their physical activity. Participants can also interact with others on Go&Grow. Go&Grow was piloted with 18 dementia caregivers (ages 22-70) over a six-week period. Multilevel modeling results showed that weeks with more Go&Grow usage were associated with more steps and more social contact. Days with more social contacts on Go&Grow were associated with more physical activity. Participants reported they were able to manage their distress better at posttest compared to the pretest. The discussion will highlight study limitations as well as implications for technology-based game and intervention design for caregiver well-being. Part of a symposium sponsored by Technology and Aging Interest Group.

